# Melatonin Maintains Fruit Quality and Reduces Anthracnose in Postharvest Papaya via Enhancement of Antioxidants and Inhibition of Pathogen Development

**DOI:** 10.3390/antiox11050804

**Published:** 2022-04-20

**Authors:** Silin Fan, Qian Li, Shujie Feng, Qiumei Lei, Farhat Abbas, Yulin Yao, Weixin Chen, Xueping Li, Xiaoyang Zhu

**Affiliations:** Guangdong Provincial Key Laboratory of Postharvest Science of Fruits and Vegetables, Engineering Research Center for Postharvest Technology of Horticultural Crops in South China, Ministry of Education, College of Horticulture, South China Agricultural University, Guangzhou 510642, China; 1575644390@foxmail.com (S.F.); liqian9808@163.com (Q.L.); sjief@scau.edu.cn (S.F.); 1198960312@foxmail.com (Q.L.); farhatmerani@yahoo.com (F.A.); 793193660@foxmail.com (Y.Y.); wxchen@scau.edu.cn (W.C.); lxp88@scau.edu.cn (X.L.)

**Keywords:** papaya, melatonin, ripening, reactive oxygen species, antioxidants, disease resistance

## Abstract

Papaya fruit is widely grown in tropical regions because of its sweet taste, vibrant color, and the huge number of health benefits it provides. Melatonin is an essential hormone that governs many plants′ biological processes. In the current study, the impact of melatonin on fruit ripening and deterioration in postharvest papaya fruit was explored. An optimum melatonin dose (400 μmol L^−1^, 2 h) was found to be effective in delaying fruit softening and reducing anthracnose incidence. Melatonin enhanced antioxidant activity and decreased fruit oxidative injury by lowering superoxide anion, hydrogen peroxide, and malondialdehyde content by enhancing the enzymatic and non-enzymatic antioxidants, and by improving the antioxidant capacity of papaya fruit. Melatonin increased catalase, ascorbate peroxidase, NADH oxidase, glutathione reductase, polyphenol oxidase, superoxide dismutase, and peroxidase activity, as well as induced total phenol, total flavonoid, and ascorbic acid accumulation. Melatonin also enhanced the activity of defense-related enzymes, such as chitinase, 4-coumaric acid-CoA-ligase, and phenylalanine ammonia lyase, while it repressed lipid metabolism. Additionally, melatonin inhibited the development of anthracnose in vitro and in vivo. These findings suggest that exogenous melatonin application improves papaya fruit quality by boosting antioxidant and defense-related mechanisms.

## 1. Introduction

Melatonin (*N*-acetyl-5-methoxytryptamine) is a crucial multifunctional signaling molecule ubiquitously distributed across numerous plant species [1]. Melatonin has demonstrated a promising role in the transportation and storage of horticultural crops as a safe and nontoxic substance. It has also been proved to be beneficial in terms of guaranteeing optimum agricultural output and food safety while remaining environmentally friendly. Exogenous melatonin application has been demonstrated in several research studies to delay postharvest ripening of several horticultural crops, such as peach [2], pear [3], apples [4], guava fruit [5], pomegranate [6] and banana [7,8]. Furthermore, melatonin has been shown to be beneficial against cold stress in a variety of crops, including broccoli [9], pomegranate [10], and tomatoes [11]. Melatonin application has been demonstrated to improve disease resistance and lower the deterioration frequency of several fruits during storage, such as lychee [12], strawberry [13] and peach [2].

Fruit ripening is frequently associated with the formation and emission of reactive oxygen species (ROS), which function as a ripening stimulant by oxidizing proteins and membrane lipids [14]. Exogenous melatonin can inhibit ROS accumulation in certain fruits by inducing antioxidant mechanisms, which delay fruit ripening [4]. Melatonin treatment can also elevate endogenous melatonin contents during fruit ripening, influencing fruit quality and shelf life of fruits such as pears [15] and bananas [7].

ROS are well known to play a significant role in fruit senescence during postharvest storage. Excessive ROS promotes fruit senescence by producing oxidative damage to cell processes, structures, and genetic macromolecules [16]. To minimize oxidative damage, many plants have developed several enzymatic and non-enzymatic antioxidative strategies [17]. Melatonin application can stimulate enzymatic and non-enzymatic antioxidant properties in various fruit varieties [18] to scavenge ROS, which assists in delaying fruit ripening and maintaining fruit quality. 

Papaya (*Carica papaya* L.), one of the most significant economic fruits from the tropics and subtropics, has a high nutritional, economic, and medical importance [19]. Papaya fruit, like any other climacteric fruit, ripens quickly after harvest and rapidly softens and decays during storage and transportation, resulting in a short shelf life and severe economic losses [20]. Anthracnose is a common postharvest disease that affects a variety of tropical and subtropical fruits, including papaya, mango, banana, apple, passion fruit, guava, grapes, and citrus [21]. Papaya anthracnose is caused by *Colletotrichum* spp. fungi, which is typically caused by quiescent infections during the flowering stage that stay quiescent until ripening and senescence [21,22]. Fruit quality dramatically decreases after the observation of anthracnose symptoms, which are small well-defined dried pink spots on the surfaces observed, and later changing into dark brown to black-colored sunken lesions [23]. Several investigations have been performed to investigate the reduction of anthracnose in papaya fruit using synthetic fungicides [23], hot water treatment [24], and a combination of hot water dipping and chitosan coating, among other methods, which limited anthracnose in papaya fruit and serve as an effective approach to combat papaya anthracnose during postharvest to prolong ripening process [25].

The current study intends to investigate a new, safe, and ecologically acceptable method of preserving papaya fruit. Melatonin’s effects on papaya fruit ripening and quality throughout postharvest storage periods were investigated. The results will provide useful information for further application of melatonin on papaya fruit during postharvest storage, transportation, and marketing.

## 2. Materials and Methods

### 2.1. Plant Materials and Treatments

The papaya fruit was collected at the color break stage from a small commercial farm in Qingyuan City, South China. Fruit of uniform shape and without blemishes were chosen, cleansed, and soaked in a sodium hypochlorite solution (0.2%) for 10 min before being subjected to various doses of melatonin (Sangon Biotech, Shanghai, China) solution. Melatonin stock solution was prepared by dissolving it in ethanol and then gradually diluting the solution to the following treatment doses based on the preliminary experiment: 0 μmol L^−1^, 100μmol L^−1^, 400 μmol L^−1^, and 800 μmol L^−1^. After 2 h of immersion treatment, the fruit was air-dried at room temperature, packed in unopened plastic bags of 0.02 mm in thickness, and kept at 25 ± 1 °C with a humidity of 70–80%. The assay was performed in triplicates, and each treatment had 90 fruits. The fruit was assessed for ripening signs on a regular basis, and pulp samples were obtained in the center of the fruit at 0, 3, 5, 7, 9, and 13 days following treatment.

### 2.2. Fruit Firmness, Respiration Rate, and Ethylene Production

Fruit firmness, ethylene production, and respiration rate were determined as described previously [26]. Fruit firmness was determined at five time points for three fruits per replicate, expressed in Newtons. Five fruits per replication were weighed and placed in a 2.5 L plastic container for 2 h at 25 °C before sample collection for the respiration rate and ethylene production assays. The replicate samples of 1 mL of headspace gas were removed and inserted into gas chromatography (Model GC-17A; Shimadzu Co., Kyoto, Japan) for a determination of ethylene, and for CO_2_ determination, a gas chromatography model G3900 (Shimadzu, Kyoto, Japan) was used. The rates of ethylene production were measured in ng kg^−1^ s^−1^, while the rates of CO_2_ production were measured in mg kg^−1^ s^−1^.

A Chromameter-2 reflectance colorimeter (Minolta, Osaka, Japan) coupled with a CR-300 reading head was used to determine the color of each fruit at five different locations around the equatorial zone. Lightness (L*), hue angle (*h°*), and chroma (C*) were calculated from the results.

### 2.3. Assessment of Fruit Disease

The fruit natural disease occurrence and severity during storage was monitored on a regular basis as described earlier [24], which graded the lesion dimension and number on a scale of 0 to 8:0 = without lesion; 1 = 1–5 mm lesion diameter = <20; 2 = 6–15 mm lesion diameter; 3 = lesion proportion = <1/16; 4 = lesion proportion = <1/8; 5 = 1/8 = <lesion proportion = <1/4; 6 = 1/4 = <lesion proportion = <1/2; 7 = 1/2 = < lesion proportion = <2/3; 8 = decay completely. The disease symptom of pink spots on the surfaces was observed when fruit start ripening, and later changing into black-colored sunken lesions. The disease index (DI) was calculated according to (DI) = [∑(disease grade × number of fruit with disease/(total number of fruit × maximal disease grade)] × 100.

The incidence of disease was estimated by dividing the number of diseased fruits by the total number of fruits.

The fruit commodity rate was evaluated on the disease severity, where fruits with disease grade >2 were considered to be without commodity value. The commodity rate was determined by dividing the number of fruits with commodity worth by the total quantity of fruits.

### 2.4. Measurement of ROS and Malondialdehyde

The malondialdehyde (MDA), superoxide anion radical (O_2_^−^), and hydrogen peroxide (H_2_O_2_), contents were measured using UV-vis spectrophotometry using the kit from Suzhou Keming Biological Company (No.SA-2-G, No.H_2_O_2_-2-Y, No.MDA-2-Y, Jiangsu, China), following the manufacturer’s protocol. The absorbance at 530 nm was determined and computed to obtain the O_2_^−^ concentration, which was given in mmol kg^−1^ units. The absorbance value was measured at 415 nm, with units denoted as mmol kg^−1^ for the H_2_O_2_ level. MDA contents were determined by measuring the absorbance value at 532 nm, with units expressed as μmol kg^−1^.

### 2.5. Measurement of Enzyme Activity and Metabolite Content

#### 2.5.1. Determination of Antioxidant Contents and Enzyme Activity

UV-vis spectrophotometry was employed to assess the ascorbate peroxidase (APX), polyphenol oxidase (PPO), catalase activity (CAT), glutathione reductase (GR), peroxidase (POD), superoxide dismutase (SOD), and total antioxidant capacity (T-AOC) via using various kits obtained from Suzhou Keming Biological Company (No.CAT-2-W, S. A NOX micro-assay kit Suzhou, obtained from Geruisi Biological Company, was used to test the NADH oxidase (NOX) activity (No.G0803W, Jiangsu, China)). The absorbance readings of SOD were obtained at 450 nm and presented in U kg^−1^, while the absorption spectrum for the CAT activity was evaluated at 240 nm, and the quantities were given as mmol min^−1^kg^−1^. Similarly, the absorbance readings for GR activity were assessed at 340 nm, and the quantities are represented in nmol min^−1^ g^−1^, whereas the absorption spectrum for APX activity was obtained at 290 nm, and the values are given in μmol min^−1^ kg^−1^.

POD activity was measured at 470 nm, and the units were denoted in U kg^−1^. The absorbance value was determined at 525 nm for PPO, and the change in absorbance at 525 nm by 0.01 increments per minute was an enzyme activity unit (U). The units are expressed in U kg^−1^. The absorbance value was determined at 593 nm for T-AOC, and the units are expressed in mmol Trolox^−1^kg^−1^. For NOX activity, the absorbance value was measured at 340 nm to obtain NADH oxidation. The units are expressed in U kg^−1^.

#### 2.5.2. Measurement of Phenylpropanoid Metabolism-Related Enzyme Activities

The efficiency of three major enzymes (4-coumaric acid: coenzyme A ligase; 4CL, cinnamic acid-4-hydroxylase activity; C4H, and phenylalanine ammonia lyase; PAL) responsible for phenylpropanoid metabolism was analyzed using enzyme activity kits purchased from Suzhou Geruisi Biological Company (No. G1003W and NoG1001W, Jiangsu, China) following the manufacturer’s instructions.

#### 2.5.3. Analysis of Ascorbic Acid (ASA), Total Phenols and Flavonoids Contents

Total flavonoid content was measured using a kit titled “total flavonoid content” acquired from Suzhou Keming Biological Company in Jiangsu, China, per the package recommendations, and absorbance was recorded at 510 nm, with values denoted as g kg^−1^.

The ascorbic acid (ASA) contents were determined using the ASA content kit (Suzhou Keming Biological Company, Suzhou, China), following the manufacturer’s protocol. The absorbance was calculated at 420 nm, and the units are represented in g kg^−1^.

Total phenolic (TP) contents were obtained using a TP content micro-assay kit (Suzhou Geruisi Biological Company, Suzhou, China), per the manufacturer’s suggestions. The absorption value was recorded at 760 nm and the units are expressed in g kg^−1^.

#### 2.5.4. Determination of Enzyme Activity Related to Fruit Stress Resistance

Chitinase (CHI) activity was determined using a CHI content micro-assay kit (No. GO546W, Suzhou Geruisi Biological Company, Jiangsu, China). The absorption at 585 nm was measured, and the units were expressed in g min^−1^kg^−1^.

The β-1,3-glucanase (β-1,3-GA) activity was assayed by a β-1,3-GA activity kit (No.GA-1-Y, Suzhou Keming Biological Company, Suzhou, China). The absorption at 550 nm was detected, and the units were expressed in g min^−1^kg^−1^.

#### 2.5.5. Analysis of Fruit Membrane Lipid Metabolism-Related Enzyme Activity

The lipase (LPS) and lipoxygenase (LOX) activity were measured using a kit obtained from Suzhou Geruisi Biotechnology Co., Ltd. (Suzhou, China), following the manufacturer’s recommendation. For LPS activity, the absorption was calculated at 405 nm, and the units are shown in mmol min^−1^ kg^−1^. For LOX activity, the absorbance was recorded at 234 nm, and the units are denoted in U kg^−1^.

### 2.6. Effects of Melatonin on Mycelia Growth In Vitro

The papaya anthracnose was caused by *Colletotrichum gloeosporioides* Penz. and *Colletotrichum brevisporum* (*C. brevisporum*) in China [27]. The *C. brevisporum* strain was provided by the pathology laboratory of the school, which was isolated from the papaya fruit harvested in the Guangzhou area, identified by morphological characteristics and sequence analysis (Supplementary Figures S1 and S2, Supplementary Table S1, Supplementary Data). The anthracnose was cultured in a PDA (potato, dextrose, and agar) medium at 28 °C. Aliquots of melatonin stock solution were added to the PDA medium to generate final concentrations of 0 μmol L^−1^, 400 μmol L^−1^, 600μmol L^−1^, 800 μmol L^−1^,1000 μmol L^−1^ and 1200 μmol L^−1^, respectively. The PDA medium was poured immediately into individual Petri dishes. A disk (5 mm diameter) of *C. brevisporum*, taken from the periphery of actively growing cultures using a sterile cork borer, was placed in the middle of each plate. The prepared plates were incubated at 28 °C for 8 days and the assay was performed in triplicate. Fungi growth was measured using a caliper to determine the diameter of radial growth.

### 2.7. In Vivo Inhibitory Efficacy of Melatonin on C. brevisporum

As our results found, 400 μmol L^−1^ melatonin of treatment showed the best effect on maintaining fruit quality, then this concentration treatment was selected for the in vivo assay. Fruits were treated with 0 and 400 μmol L^−1^ melatonin solution and stored at 25 ± 2 °C for three days. Then, all fruits were ripened via immersion in 800 μmolL^−1^ ethephon solutions for 1 min and air-dried at room temperature. The fruits were wounded using a sterile nail (1 mm wide and 1 mm deep) at three different points in each fruit and further inoculated with either 5 μL of *C. brevisporum* spore suspension (5 × 10^5^ spores mL^−1^) or water at each wound. Treated fruits were packaged in 0.02 mm thicker open plastic bags to ensure high humidity (95%) and kept at 25 °C. The diameter of the disease lesions was measured 24 h after each treatment when small pink spots on the surfaces were observed and later changed into white-grayish discoloration of sunken lesions. All treatments were performed in triplicate, with at least 15 fruits in each biological replicate.

### 2.8. Statistical Analysis

The data was analyzed using SPSS17.0 and Excel 2019 to calculate means and standard errors (SE). Each assay was performed in triplicate and SigmaPlot 12.0 was used to plot the figures. Duncan’s multiple range test was used to find significant differences between treatment groups for each data set (*p* < 0.05). The least significant difference (LSD) was used for the analysis of variance. The results are expressed as means ± SE.

## 3. Results

### 3.1. Effect of Melatonin on Fruit Ripening

**Figure 1:**
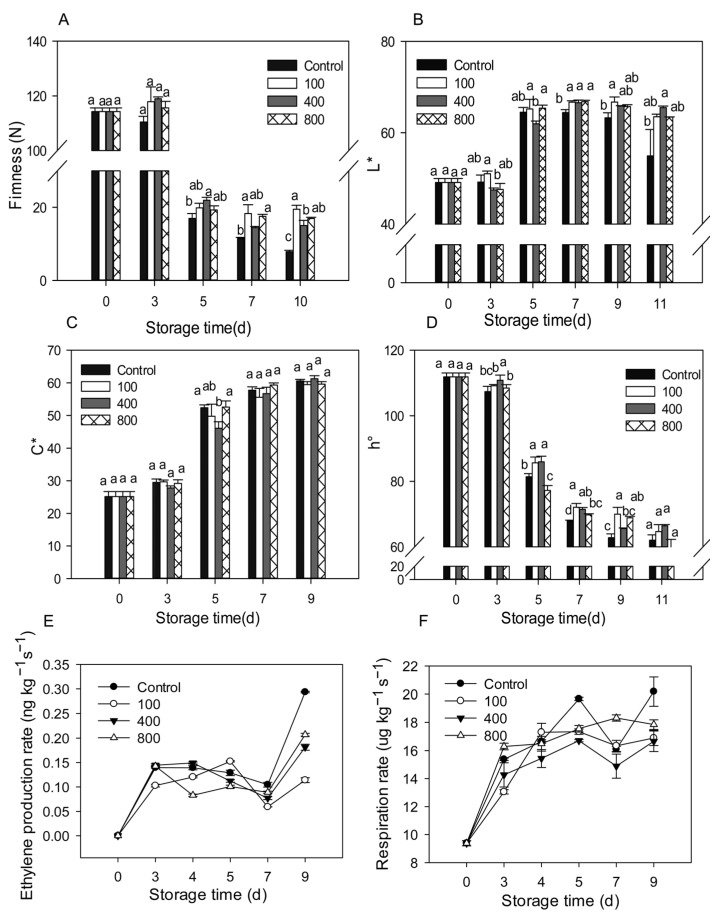
Effects of melatonin applications on fruit physiology. (**A**) Impact of melatonin treatments on papaya fruit firmness. (**B**,**C**) The effect of melatonin treatment on the chromaticity L* (**B**), C* (**C**), h° (**D**) of papaya fruit. (**E**) The effect of melatonin treatment on ethylene production rate in papaya fruits. (**F**) The effect of melatonin treatment on the respiration rate in papaya fruits. Each data point indicates the mean standard deviation (*n* = 3). Different letters denoted substantial changes at the 5% level. To compare significant effects at the 5% level, the least significant differences (LSDs) were determined. “100”, “400” and “800” indicated the “100 μmolL^−1^”, “400 μmolL^−1^” and “800 μmolL^−1^” melatonin treatments.

Upon harvest, papaya fruit will ripen and soften rapidly. Fruit firmness declined considerably from day 3 to day 5 and then remained low, as illustrated in Figure 1A. Compared to control treatment, all melatonin treatments delayed fruit firmness decrease, maintaining higher fruit firmness during the later storage (Figure 1A). As the fruit ripens, the fruit peel color gradually turns from green to yellow. The fruit peel color is mostly assessed using L*, C*, and *h°* values, which represent the brightness, color saturation, and hue of the peel, respectively. The L* value for the control group rose during fruit ripening and declined slightly during late storage, as seen in Figure 1B. During the later storage period, the melatonin treatments retained greater L* levels than the control group (Figure 1B). The C* value of the fruit increased during ripening, but no significant differences were observed between the groups (Figure 1C). During fruit ripening, the *h°* value gradually dropped. During late storage, the melatonin treatments prevented the decrease of the h° value and showed a greater *h°* value than the control fruit (Figure 1D).

Ethylene production increased rapidly after harvesting and reached its peak on day 3 after harvest. Levels then decreased slightly after that but dramatically increased between day 7 and day 9. Treatment with 100 μmol L^−1^ melatonin prolonged the ethylene peak by about two days and suppressed ethylene synthesis during late storage. Melatonin treatments of 400 and 800 μmol L^−1^ did not affect the ethylene production peak, but reduced ethylene production after day 4, especially during the late storage period (Figure 1E). All melatonin treatments reduced ethylene production during the later storage. Fruit respiration accelerated in tandem with fruit ripening, with a maximum on day 5, then declined, then rose again on day 7 (Figure 1F). All melatonin treatments showed similar respiration rates compared to the control group but had lower respiration on day 5 and day 9. The melatonin treatments repressed the fruit respiration rate during the later storage.

### 3.2. Effects of Melatonin on the Development of Fruit Disease

**Figure 2:**
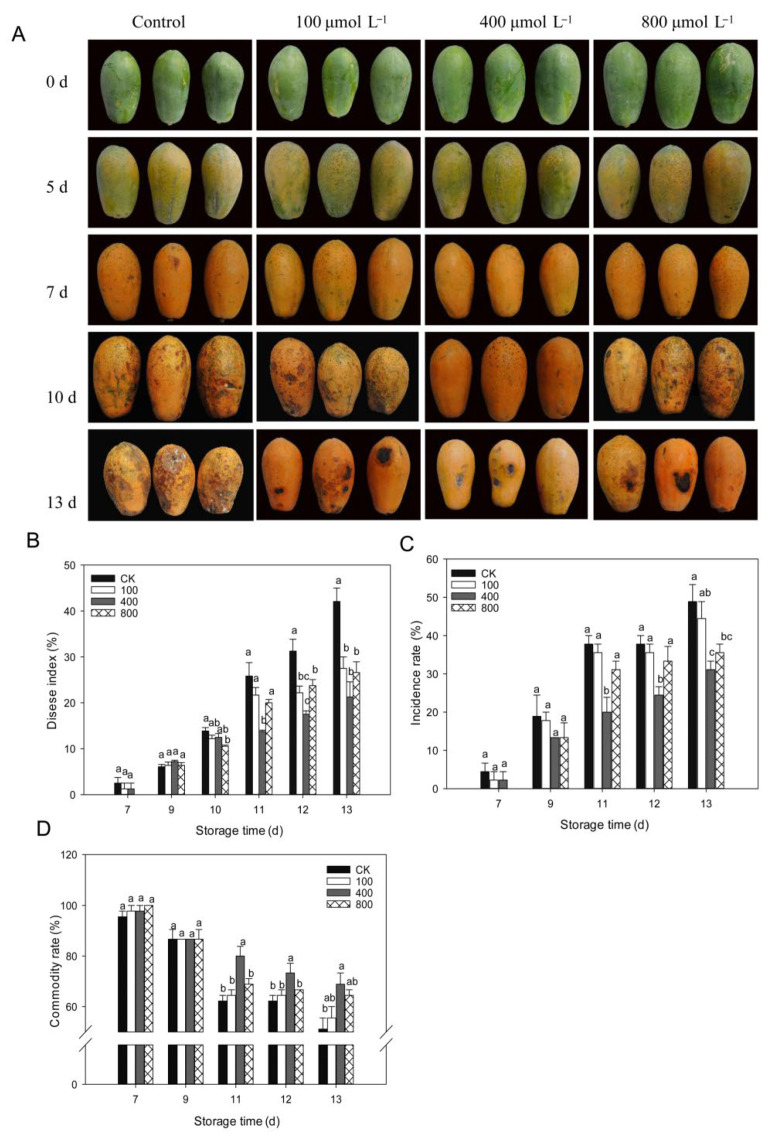
Impact of melatonin application on fruit disease incidence. (**A**) Pictorial view of papaya fruits during various melatonin applications. (**B**) Fruit disease index. (**C**) Fruit disease incidence. (**D**) Fruit commodity rate. Papaya fruit were stored at room temperature (25 ± 1 °C) after treatment. The data are shown as mean ± SE (*n* = 3) and different letters represent the significant differences at the 5% level. CK: control group, “100”, “400” and “800” indicated the “100 μmolL^−1^”, “400 μmolL^−1^” and “800 μmolL^−1^” melatonin treatments.

Anthracnose is, by far, the most frequent disease that affects papaya during the postharvest period. As shown in Figure 2, papaya fruit ripens rapidly after being harvested, and disease symptoms are observed starting from day 7 after harvest (Figure 2A). On day 10, following harvest, serious disease symptoms were noticed, and melatonin treatments inhibited disease progression in papaya fruit, particularly in the 400 μmol L^−1^ melatonin treatment. As demonstrated in Figure 2B, the disease index rapidly increased during fruit ripening, and melatonin treatments lowered the disease index and showed a reduced disease index than control fruit during late storage, particularly in the 400 μmol L^−1^ melatonin treatment. A similar finding was shown for disease incidence (Figure 2C), where the 400 μmol L^−1^ melatonin treatment had the lowest disease incidence rate compared to other treatment groups. The fruit commodity rate decreased during fruit ripening due to the development of disease. Melatonin-treated fruit showed a higher commodity rate than the control fruit, and the 400 μmol L^−1^ melatonin treatment group showed the highest commodity rate compared to other treatments (Figure 2D).

### 3.3. Effect of Melatonin on the Metabolism of ROS

**Figure 3:**
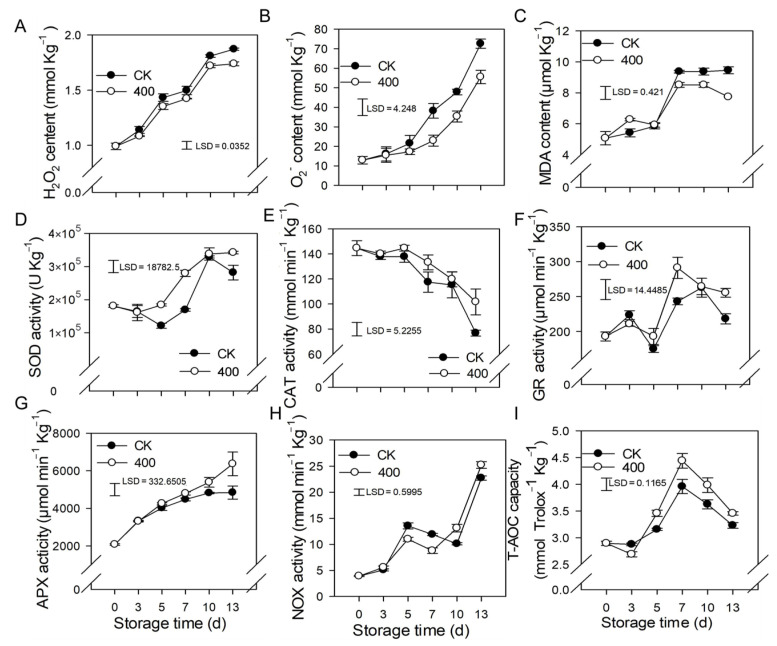
Effects of melatonin on the metabolism of reactive oxygen species in papaya fruit. (**A**) Impact of melatonin treatment on H_2_O_2_ (**B**), and O_2_^−^ contents in papaya fruit. (**C**), the effect of melatonin treatment on MDA (**D**), SOD (**E**) CAT (**F**) GR (**G**) APX (**H**) NOX (**I**) and T-AOC capacity in papaya fruits. Each data point represents the mean ± SE (*n* = 3). Least significant differences (LSDs) were calculated to compare significant effects at the 5% level. CK: control group, “400” indicated the “400 μmolL^−1^” melatonin treatments.

When plants are exposed to unfavorable conditions, hydrogen peroxide (H_2_O_2_) and O^2−^ are major ROS. In Figure 3, the H_2_O_2_ and O^2−^ content of papaya steadily increased with fruit ripening in the control. Similar results were observed in the 400 μmol L^−1^ melatonin-treated fruit, but H_2_O_2_ and O_2_^−^ contents were significantly lower than in the control group (Figure 3A,B). 

Malondialdehyde (MDA) is the end result of lipid peroxidation in cells, and its quantity in plants serves as an indication of lipid oxidation levels. The MDA content rose in line with fruit ripening, and increased considerably from day 5 to day 7, but remained relatively steady during late storage (Figure 3C). Melatonin treatment decreased MDA levels after late storage, which was likewise lower than the control group, implying a lower degree of peroxidation in the papaya fruit.

The results showed that SOD, APX, GR, and NOX activity increased throughout fruit ripening (Figure 3D,F–H). During the first three days, there was no significant difference between the control and treatments, however, three days after storage, melatonin treatment boosted the activity of SOD, APX, GR, and NOX compared to the control treatment. (Figure 3D,F–H). The CAT activity declined throughout fruit ripening, whereas melatonin treatment not only delayed but also improved the CAT activity related to the control group (Figure 3E). 

The total antioxidant capacity of papaya fruit is comprised of several antioxidants and antioxidant enzymes (T-AOC). As demonstrated in Figure 3I, the T-AOC concentration gradually increased, peaked on day 7, and subsequently declined during the late storage period (Figure 3I). Melatonin treatment stimulates the production of T-AOC content, resulting in fruit with higher T-AOC content than the control group. 

### 3.4. Effects of Melatonin on Lignin Biosynthesis Enzymes and Defense-Related Substances

**Figure 4:**
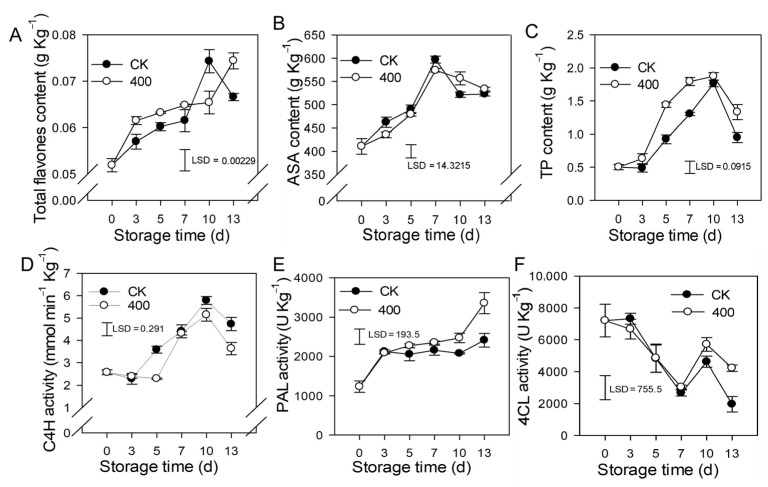
Effects of melatonin application on lignin biosynthesis enzymes activity and other defense-related substances in papaya fruit. (**A**) Effect of melatonin application on total flavones (**B**) ASA (**C**) TP (**D**), C4H (**E**) PAL (**F**) 4CL activity. The experiments were carried out in triplicate and each data point shows the mean ± SE (*n* = 3). To compare significant effects at the 5% level, least significant differences (LSDs) were determined. CK: control group, “400” indicated the “400 μmolL^−1^” melatonin treatments.

Flavonoids are secondary metabolites found in plants that have great antioxidant properties and modify the color and flavor of the fruit. The results revealed that total flavones content, ASA, and TP increased during fruit ripening, but slightly decreased in the late storage of the control fruit (Figure 4A–C). Melatonin treatment induced the accumulation of total flavones and TP, which was higher than what was found in the control fruit. There was no substantial difference in ASA content between the melatonin treatment and the control (Figure 4B).

C4H is a major enzyme in the phenylpropane metabolic pathway that governs the formation of flavonoids and other metabolites. The results indicate that activity of C4H was increased during fruit ripening, and slightly decreased during late storage. Melatonin treatment reduced C4H activity, and was lower than the control group on day 5 and day 13, but not significantly lower on other days (Figure 4D). PAL activity gradually increased during fruit ripening, and melatonin treatment induced its activity. PAL activity was higher in melatonin-treated fruit than in the control after late storage, although no significant difference was seen during the first 7 days (Figure 4E). The activity of 4CL reduced throughout fruit ripening while increasing on the tenth day. During the first 10 days of storage, there was no significant difference between the melatonin treatment and the control, however, on day 13, the melatonin-treated fruit had higher 4CL activity than the control fruit (Figure 4F). 

### 3.5. Effect of Melatonin on Phenolic, Lipid Metabolism, and Other Defense-Related Enzymes

**Figure 5:**
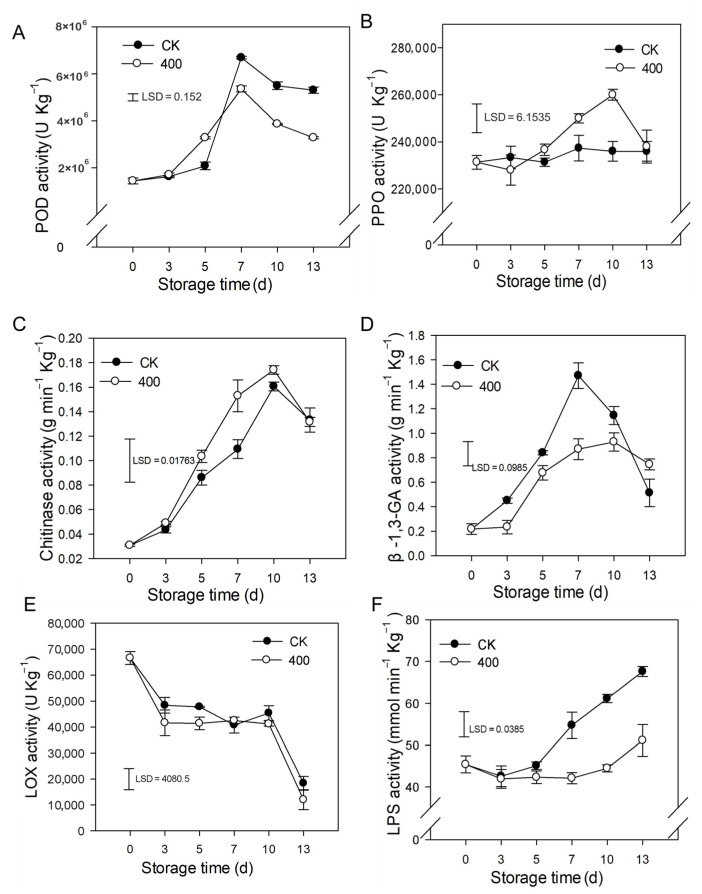
Impact of melatonin applications on phenolic, lipid metabolism, and other defense-related-enzymes activities in papaya fruit. (**A**) Effect of melatonin treatment on POD (**B**) PPO (**C**) CHI (**D**) β-1,3-GA (**E**) LOX and (**F**) LPS activity. Each data point indicates the mean SE (*n* = 3), and LSD was computed to compare significant effects at the 5% level. CK: control group, “400” indicated the “400 μmolL^−1^” melatonin treatments.

The results indicate that POD activity rapidly increased and peaked on day 7 and slightly decreased during fruit ripening. Similarly, POD activity was lower in melatonin-treated fruit compared to control fruit after late storage (Figure 5A). PPO activity slightly increased during the entire storage period and was enhanced by melatonin treatment. There was higher PPO activity during late storage compared to the control group (Figure 5B).

As demonstrated in Figure 5C, CHI activity rapidly increased during fruit ripening and slightly decreased thereafter (Figure 5C). Melatonin treatment boosted CHI activity, with higher observed enzyme activity than in the control group (Figure 5C). β-1,3-GA activity rose dramatically throughout fruit ripening with a maximum at day 7 and decreased afterward at the end of storage (Figure 5D). Melatonin severely decreased the β-1,3-GA activity during the storage period.

LOX activity was decreased with the fruit ripening, and no substantial difference was detected between the control and melatonin treatments (Figure 5E). Likewise, LPS activity was gradually increased during fruit ripening, and melatonin treatment severely suppressed LPS activity (Figure 5F). 

### 3.6. Effect of Melatonin on Mycelial Growth In Vitro

**Figure 6:**
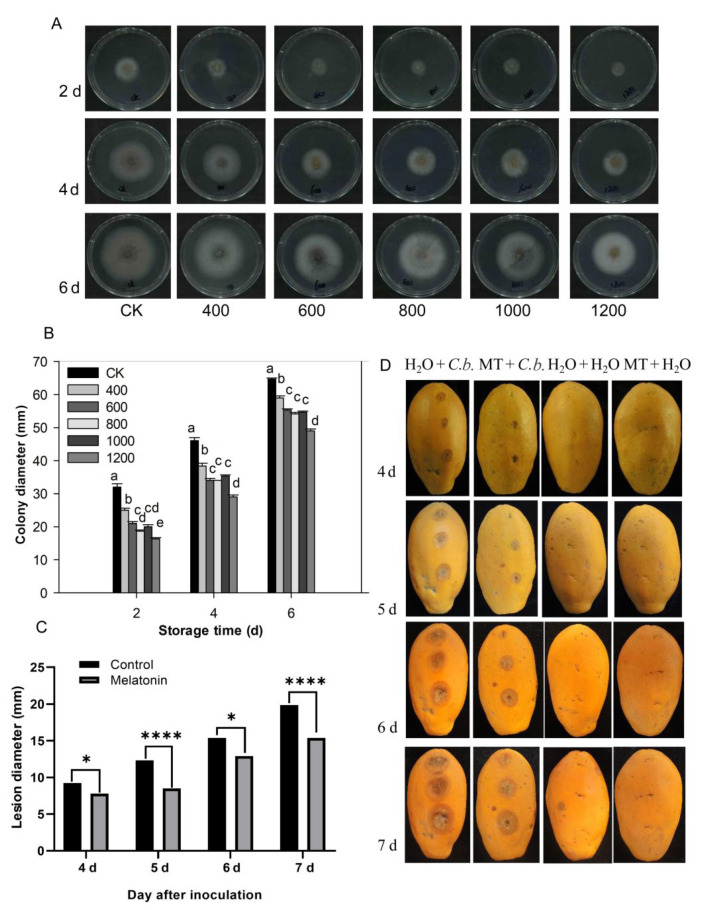
Effect of melatonin application on the development of *C. brevisporum* in vitro and in vivo. (**A**) Colony morphology of *C. brevisporum* on PDA medium with different concentrations of melatonin. (**B**) The effect of melatonin treatment on colony diameter (**C**) and lesion diameter of anthracnose. (**D**) Disease symptoms on papaya fruit after inoculation for 4, 5, 6 and 7 days. Each data point denotes the mean ± SE (n = 3). Different letters show substantial differences at the 5% level. * and **** indicated a significant difference at 0.05 and 0.001 levels, respectively. CK: control group, “400”, “600”, “800” “1000”, and “1200”, indicated the “400 μmolL^−1^”, “600 μmolL^−1^”, “800 μmolL^−1^”, “1000 μmolL^−1^” and “1200 μmolL^−1^” melatonin treatments. MT: 400 μmolL^−1^ melatonin treatment, *C.b.*: *Colletotrichum brevisporum*.

The pathogen *C. brevisporum* was provided by the pathology laboratory of the school and was used for experiments in this study. The morphological characteristics and sequence analysis of *C. brevisporum* were confirmed and shown in the Supplementary Materials file (Supplementary Figures S1 and S2, Supplementary Table S1, Supplementary Data). As shown in Figure 6, each treatment colony gradually expanded as storage time increased, and the diameters of the colonies in the control group were larger than in the melatonin treatment groups (Figure 6A,B). Mycelial growth inhibition was dose-dependent and was enhanced with an increasing concentration of melatonin. The melatonin 600, 800, and 1000 μmol L^−1^ treatment groups did not differ significantly. However, the colony diameter of the 1200 μmol L^−1^ group was smaller than in the other treatment groups (Figure 6B). These results indicate that melatonin can inhibit *C. brevisporum* growth in vitro.

### 3.7. Melatonin Treatment Inhibits the Growth of Anthracnose on Papaya Fruit

The papaya fruit stab inoculation experiments were conducted to test the effect of melatonin on anthracnose development in vivo. After 4 days of inoculation, there were visible lesions in the papaya fruit except for those inoculated with water (Figure 6C,D). As storage time increased, the lesion diameter in papaya fruit in the control treatment group (H_2_O) increased rapidly. The lesion diameter in fruit treated with melatonin also increased with increased storage time, but was overall, much smaller and grew more slowly than in the control group (Figure 6C,D). No disease symptoms were observed in fruit inoculated with H_2_O, nor in melatonin or H_2_O treated fruit (Figure 6D). These results indicate that melatonin treatment suppressed *C. brevisporum* development and anthracnose development in papaya fruit.

## 4. Discussion

Over the past few decades, various postharvest technologies have been investigated and developed to prolong the shelf life of papayas and reduce postharvest loss. It has been reported that 1-MCP [28], hot water [24], hot water dip and chitosan combination treatment [25], salicylic acid, nitric oxide, and edible coating applications [29] may prolong fruit ripening and retain quality during the postharvest period. However, most of these treatments have some problems during their implementation. For example, when choosing the right 1-MCP treatment concentration, difficulties arise as an unsuitable 1-MCP treatment will cause a “rubbery texture” [28] ripening disorder in the fruit. Hot water treatment reduced anthracnose incidence in papaya fruit, but accelerated fruit ripening [24]. Melatonin is a safe and environmentally friendly material. It has gained more attention in postharvest preservation. Various works have confirmed that melatonin application can postpone the ripening and senescence in postharvest fruit, as well as prolong their shelf life. 

In recent years, increasing literature has shown that melatonin treatment may effectively postpone fruit ripening and senescence, maintaining fruit quality [7,8]. However, there are also several reports showing that melatonin treatment accelerated fruit ripening. For example, melatonin treatment increases the lycopene content in tomatoes and enhanced the ethylene release rate [30]. Exogenous melatonin on young grapes fruit can promote the growth and ripening of grapes fruit and increase its endogenous melatonin content [31]. In our present work, the results showed that appropriate melatonin treatment can delay the papaya fruit softening, the peak of ethylene content, and respiration rate, which are consistent with most of the other studies on other fruit crops. All melatonin treatments could effectively reduce the ethylene and respiration rate during the later storage, which shows a more significant effect than the climacteric period of fruit ripening (Figure 1). Moreover, melatonin treatment also reduced fruit anthracnose incidence in papaya, which could maintain a higher commodity rate than the control group, and reduce the product loss during the shelf life. Actually, as a classic climacteric fruit, ethylene production and respiration should decrease during later storage. The increase in ethylene and respiration rate could be due to the increase of disease during the later storage. The melatonin treatment effectively reduced the disease development, resulting in a reduced ethylene and respiration rate. It seems that melatonin shows to be more effective on disease incidence than fruit ripening. Similar results were also found in fruit, such as strawberry [13] and peach [2], that melatonin application can enhance disease resistance and reduce the decay incidence. Therefore, melatonin exhibits application potential as a safe and nontoxic molecular in postharvest papaya fruit.

Melatonin treatment’s effect on fruit ripening and disease resistance was dose-dependent according to our findings. An intermediate concentration showed the best overall effect, rather than the lowest or highest concentrations. Our results showed that the intermediate concentration of 400 μmol L^−1^ melatonin treatment exhibited a better overall effect on fruit ripening and disease inhibition than either low (100 μmol L^−1^) or high (800 μmol L^−1^) concentrations. Previous work showed that 0.1 and 1.0 μM melatonin had no effect on fruit ripening, but a 100 μM melatonin treatment could effectively promote the grape berry fruit ripening [32]. In apple trees, the increased melatonin concentration at a suitable range enhanced flowering, but unsuitable high concentrations would reduce flowering [18]. Our previous work also showed that the effect of melatonin on guava fruit ripening and disease control is dose-dependent [5]. This effect may be due to signal cross-talk in fruit. Different plants have shown different levels of melatonin and vary dramatically across species, with the lowest measured level (0.1 pg g^−1^) in currant tomato (*Solanum pimpinellifolium* L.) and the highest measured level (340,000 pg g^−1^) in yarrow plant (*Achillea millefolium* L.) [33]. Different plants appear to respond differently to melatonin, and there is extensive signal cross-talk between signal pathways following melatonin treatment. Melatonin influences signal molecules like ABA, SA, ethylene, IAA, GA, and NO, and endogenous melatonin in plants regulates fruit ripening and stress response [1]. Thus, the effects of melatonin application on plants can be species-dependent and dose-dependent.

Melatonin is a powerful antioxidant in plants. Fruit ripening is frequently accompanied by an increase in ROS levels, which promotes oxidative damage to membrane lipid proteins, nucleic acids, and cell activity, resulting in rapid fruit ripening [14]. To defend against oxidative damage, certain fruits have developed sophisticated enzymatic and non-enzymatic antioxidative systems to scavenge ROS [17]. Exogenous melatonin application may minimize ROS production by increasing enzymatic and non-enzymatic antioxidants in postharvest fruit [1,5]. Melatonin application has been reported to reduce the concentration of H_2_O_2_ and MDA in strawberry fruit while increasing the accumulation of total flavonoid and phenolic content, which contributes to higher antioxidant potential in fruit [34]. Melatonin application has been found to enhance the activity of CAT, APX, SOD, and POD in peach fruit, but reduce the content of MDA, O_2_^−^ and H_2_O_2_, and the activity of lipoxygenase. All of this maintains the membrane’s integrity and results in delayed postharvest senescence [2]. 

Non-enzymatic antioxidants have also been found to be induced by melatonin application, such as in phenols, GSH, AsA, flavonoids, and anthocyanins [1,33]. Exogenous melatonin has been found to enhance the polyphenol accumulation and antioxidant activity in grape berries [35]. Melatonin application has been shown to improve total anthocyanin accumulation, total phenol accumulation, and GABA channel activity in postharvest strawberry fruit, hence maintaining nutritional quality and reducing deterioration [13]. Melatonin application has been found to increase phenolic compounds by up-regulating associated gene expression, resulting in delayed fruit senescence and quality deterioration in jujube fruit [36].

In the present study, similar results were obtained, in which melatonin enhanced the activity of enzymatic antioxidants, such as the activity of SOD, CAT, GR, APX, and NADH oxidase (NOX), POD, and PPO. Melatonin also increased the concentration of non-enzymatic antioxidants like total flavonoids, total phenols (TP), and ASA, as well as the fruit’s total antioxidant capacity (T-AOC), resulting in the reduction of H_2_O_2_, O_2_^−^, and MDA contents. According to the findings of this investigation, as well as many other studies, melatonin works well as a ROS scavenger to protect the fruit from oxidative damage.

During the postharvest period, postharvest deterioration is a primary cause of postharvest losses. Although chemical fungicides can prevent most postharvest infections, their usage is limited and regulated due to increased food safety awareness. Melatonin, being a benign and safe material, is an environmentally acceptable medication and an alternate to fungicide sprays. Melatonin has been demonstrated in numerous studies to prevent postharvest disease in a variety of fruits, including apple, grape, banana, litchi, kiwifruit, plum, peach, strawberry, and tomato [1,12]. In the present study, melatonin treatment inhibited the development of anthracnose in vitro and in vivo. In the in vitro assay, different concentration melatonin treatments could inhibit the mycelial growth of *C. brevisporum.* At a suitable range of concentration, the increased melatonin concentration enhanced inhibition of mycelial growth (0–600 μmol L^−1^) but was less effective when the concentration was higher (Figure 6A,B). The in vivo assay showed that 400 μmol L^−1^ melatonin could effectively reduce disease development after artificial wounded inoculation, and better than other concentrations (Figure 2 and Figure 6C,D). The concentration difference between in vivo and in vitro assays may be due to more complicated signal cross-talk in fruit than in Petri dishes. However, the effects of melatonin on the natural infections of *C. brevisporum* may be different from the artificial wounded inoculation as used in the present study for the in vivo assay. 

Melatonin has been shown in vitro and in vivo to have antibacterial properties against many fruit diseases. Melatonin has been proven to decrease the growth of *Phytophthora infectans* in vitro and the symptoms of potato late blight in vivo [37]. Exogenous melatonin has been shown to induce the content of arecoline, improve plant disease resistance, and delay the postharvest deterioration of areca fruit through the regulation of hormone balance, glycolytic activity, and ROS level [38]. The combined application of melatonin with *Meyerozyma guilliermondii* Y-1 has been found to enhance the activity of defense-related enzymes, including SOD, CAT, POD, PPO, and PAL, as well as T-AOC, total phenolics, and lignin content, resulting in a reduction in the decay incidence and lesion diameter of gray mold in apple fruit [39]. Melatonin is also able to enhance the phenylpropanoid pathway and enhance the accumulation of disease resistance-related phenolics by activating the intermediate synthesis pathway. For example, in litchi fruit, melatonin treatment has been found to severely restrict the development of *P. litchi* in litchi fruit after inoculation. Melatonin-induced fruit disease resistance has been found to act via enhancement of the C4H, PAL, and 4CL activity, as well as through the promotion of phenolic and flavonoid accumulation [12]. In cherry tomatoes, previous studies have found that melatonin treatment increases the content of total phenols, flavonoids, and lignin via regulation of 4CL, PAL, and POD activity in the phenylpropanoid pathway [40]. Melatonin treatment has been found to reduce gray mold development caused by *B. cinerea* by inducing a ROS burst, increasing the content of endogenous salicylic acid (SA), methyl jasmonate (MeJA), and melatonin, and enhancing the activity of CHI and β-1,3-glucanase and phenylpropanoid pathway in tomato [40,41]. Melatonin has also been shown to prevent pathogen infection by promoting the formation of cell walls, lipids, and waxes in banana peels [8], however, it is quite remarkable that melatonin reduces resistance to postharvest green mold in citrus fruit by lowering the rate of defense-related reactive oxygen species [42]. Actually, in the present work, our results showed that melatonin enhanced the defense-related enzyme activity of CHI, PAL, and 4CL, and promoted the accumulation of total flavones and TP, but repressed lipid metabolism to enhance the disease resistance in papaya fruit.

## 5. Conclusions

In this study, we found that the effect of melatonin on fruit ripening and disease resistance was dose-dependent. An intermediate concentration showed the best overall effect, rather than the lowest or highest concentrations. We further discovered that melatonin application increased antioxidant capacity and decreased oxidative damage in papaya fruit. Melatonin elevated the concentration of total flavonoids, total phenols (TP), and ASA, as well as the activity of phenylpropanoid pathway enzymes such as PAL and 4CL. Melatonin application was found to significantly boost or enhance defense-related enzyme activity, such as in CHI and its suppressed lipase activity, which is involved in fat metabolism. Additionally, melatonin treatment inhibited the development of anthracnose in vitro and in vivo. Overall, our findings indicate that exogenous melatonin has the potential to promote disease resistance in papaya fruit via improving antioxidant and defense systems.

## Data Availability

The data is contained within the article and Supplementary File.

## Supplementary Materials

The following supporting information can be downloaded at: https://www.mdpi.com/article/10.3390/antiox11050804/s1, Figure S1: Morphological characteristics of the pathogen; Figure S2: The phylogenetic tree generated by the combination of *ITS* region, *GAPDH* and *ACT* genes from different *Colletotrichum spp*., Table S1: Primers used in present work; Supplementary Data: Gene sequence of *ITS, GAPDH* and *ACT*. Reference [43] is cited in the supplementary materials.
