# Supplementary material for: Melatonin Maintains Fruit Quality and Reduces Anthracnose in Postharvest Papaya via Enhancement of Antioxidants and Inhibition of Pathogen Development
Source: Antioxidants (Basel). 2022 Apr 20;11(5):804. doi: 10.3390/antiox11050804 (PMC9137572; doi:10.3390/antiox11050804)
Supplement: Supplementary file 1 [file antioxidants-11-00804-s001.zip › antioxidants-1651784-supplementary.pdf]

Supplementary Figures

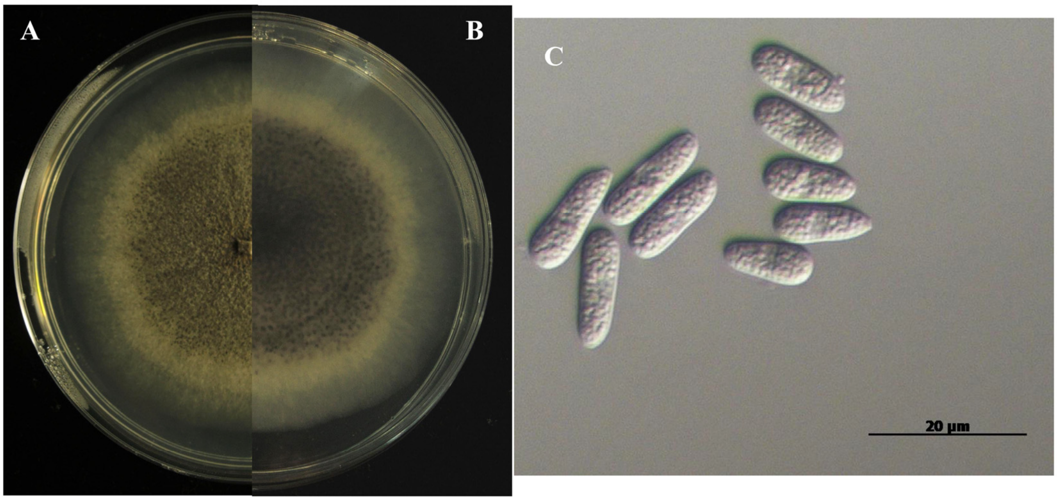

Figure S1. Morphological characteristics of the pathogen. A-B, Conony on PDA for 7 days post inoculation, upper and reverse side; C, Conidia.

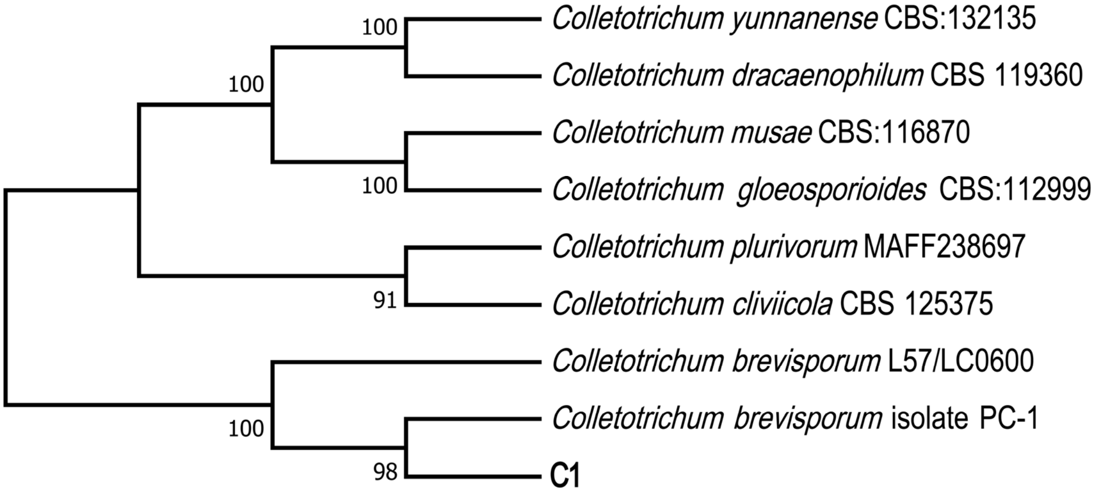

Figure S2. The phylogenetic tree generated by combination of *ITS* region, *GAPDH* and *ACT* genes from different *Colletotrichum spp.*. C1 indicted the isolation used in the present work. The accession numbers of *ITS, GAPDH* and *ACT* from different *Colletotrichum spp.* Strains were used as described in Damm et al. (2019). The sequences were extracted from NCBI. The cloned gene sequences were blasted in NCBI database, and all these sequences showed 100% identity with the corresponding gene sequences of different *Colletotrichum brevisporum* strains, with different coverage. The phylogenetic tree was constructed by MEGA 7 using the three

sequence datasets and other corresponding sequences from different *Colletotrichum spp*., by the Neighbour-Joining bootstrap with Maximum Likelihood distances (10000 replicates).

Supplementary Table S1 Primers used in present work.

Genes	Primer name	Primer sequence（5’-3’）	Length of sequence（bp）
ACT	ACT-521	ATG TGC AAG GCC GGT TTC GC	281
	ACT-783	TAC GAG TCC TTC TGG CCC AT	
GAPDH	GDF	GCC GTC AAC GAC CCC TTC ATT GA	252
	GDR	GGG TGG AGT CGT ACT TGA GCA TGT	
ITS	ITS4	TCC TCC GCT TAT TGA TAT GC	614
	ITS5	GGA AGT AAA AGT CGT AAC AAG G	

Supplementary data: Gene sequences of *ITS*, *GAPDH* and *ACT*

ITS
TTCCCCCCCCCCCCCCCCCCTCAAGCTTAAGTTCAGCGGGTATTCCTACCTGATCCGAGGT
CAACCTTGATAAATTGGGGGGTTTTACGGCAGGAGTCCCTCCGGATCCCAGTGCGAGGTGG
TATGTTACTACGCAAAGGAGGCTCCGGGAGGGTCCGCCACTGTATTTCGGGGCCTACGGAA
GCCGTAGGGCCCCAACGCCAAGCGGTGCTTGAGGGTTGAAATGACGCTCGAACAGGCATG
CCCGCCAGAATGCTGGCGGGCGCAATGTGCGTTCAAAGATTCGATGATTCACTGAATTCTG
CAATTCACATTACTTATCGCATTTCGCTGCGTTCTTCATCGATGCCAGAACCAAGAGATCCGT
TGTTAAAAGTTTTGATTATTTGCTTGTACCACTCAGAAGAGACGTCGTTGCAATAGAGTTTA
GTGGTCCGCCGGCGGGTGGGGCGCGCCGAGAAGGCGGCTCCGGGCGCCGGAGCTGGGCT
CCGGCTGCCCGCCGAGGCAACGTTTGAGGTATGTTCACAAAGGGTTATAAGGAGCGGTAAC
TCGATAATGATCCCTCCGCTGGTTCACCAACGGAGACCTTGTGTTTTTTTTTTTTTCACACA
A

GAPDH
TTTGCCGTCATTCGCCCCTTCATTGAGACCAAGTACGCTGTGAGTAGCACCTCTCCaAGCTC
GCCACGCCCGCCACCCCTCAGTCGCGAACGTCAGCTCTAGCTGTCGATAACATGCGAAATC
AATCAAGCTCCAATGCCGTGAGCGATTGATGGGGTCGGCGTGGTGGGGTTGAATATCATGC
AAAGGACTATGGTCGCTGACACGCCATCCGCAGGCCTACATGCTCAAGTCGAAACTCCACC
CAAGGAT

ACT
TGTTATGTGCAAGccCGGTTTCGCCGGTGacGAtgcgccCAGAGCTGTCTTCcgtaAGTCTTCCCG

CCTGCAGACCGCAATCTCGCCCCGTCAGGGGGCATCGAGATTTGCGGCCGGCTTCCGCTCG
CACACTTGGATGCTGACAGCTTCGCAGCCTCCATTGTTGGTCGCCCTCGCCACCATGGGTAT
GTTTCCTCTCGCCGCGGCAATTTCTCGCGTTTCCGTCGCCTCCTAACAAATgggcagtatcatGATT
GGTATGGGCCAGAVGGGACTCGTAA

Reference

1. Damm, U.; Sato, T.; Alizadeh, A.; Groenewald, J.Z.; Crous. P.W. The *Colletotrichum dracaenophilum*, *C. magnum* and *C. orchidearum* species complexes. *Stud. Mycol.* **2019**, *92*, 1–46. https://doi.org/10.1016/j.simyco.2018.04.001.
